# Development of evaluation system for cerebral artery occlusion in emergency medical services: noninvasive measurement and utilization of pulse waves

**DOI:** 10.1038/s41598-023-30229-3

**Published:** 2023-02-27

**Authors:** Takuma Shimada, Kazumasa Matsubara, Daisuke Koyama, Mami Matsukawa, Miho Ohsaki, Yasuyo Kobayashi, Kozue Saito, Hiroshi Yamagami

**Affiliations:** 1 Faculty of Science and Engineering, Doshisha University, Kyoto, Japan; 2 Department of Neurology, Nara Medical University, Nara, Japan; 3 Department of Stroke Neurology, National Hospital Organization Osaka National Hospital, Osaka, Japan

**Keywords:** Health care, Engineering

## Abstract

Rapid reperfusion therapy can reduce disability and death in patients with large vessel occlusion strokes (LVOS). It is crucial for emergency medical services to identify LVOS and transport patients directly to a comprehensive stroke center. Our ultimate goal is to develop a non-invasive, accurate, portable, inexpensive, and legally employable in vivo screening system for cerebral artery occlusion. As a first step towards this goal, we propose a method for detecting carotid artery occlusion using pulse wave measurements at the left and right carotid arteries, feature extraction from the pulse waves, and occlusion inference using these features. To meet all of these requirements, we use a piezoelectric sensor. We hypothesize that the difference in the left and right pulse waves caused by reflection is informative, as LVOS is typically caused by unilateral artery occlusion. Therefore, we extracted three features that only represented the physical effects of occlusion based on the difference. For inference, we considered that the logistic regression, a machine learning technique with no complex feature conversion, is a reasonable method for clarifying the contribution of each feature. We tested our hypothesis and conducted an experiment to evaluate the effectiveness and performance of the proposed method. The method achieved a diagnostic accuracy of 0.65, which is higher than the chance level of 0.43. The results indicate that the proposed method has potential for identifying carotid artery occlusions.

## Introduction

Cerebrovascular disease (CVD) encompasses all disorders that can lead to temporary or permanent impairment of brain function. CVD is primarily caused by ischemia or bleeding and is currently a major cause of morbidity and mortality worldwide. According to the World Health Organization, approximately 6 million people die annually from CVD^1^. In Japan, the number of such fatalities per year is approximately 110,000^2^. Ischemic strokes are typically caused by stenosis or occlusion of the cerebral arteries. Acute cerebral large vessel occlusion leads to large infarctions and can result in serious sequelae or death. Rapid reperfusion therapy, including intravenous thrombolysis and mechanical thrombectomy, can reduce disability in patients with large vessel occlusion strokes (LVOS)^3–5^. As early treatment is crucial for a successful outcome in LVOS patients, rapid transport to a comprehensive stroke center is essential. Therefore, it is necessary to develop a simple evaluation device that can easily and accurately identify LVOS in emergency medical services.

Currently, computed tomography (CT), magnetic resonance imaging (MRI), and ultrasonography are the main methods for diagnosing cerebral artery occlusions^6^. The effectiveness of mobile stroke units, which are ambulances equipped with a CT scanner, has been reported in Germany and the United States, however, they are not widely available in Japan and other parts of the world^7,8^. Additionally, portable X-ray systems can also lead to issues with exposure to harmful radiation and high costs. Therefore, a safe, inexpensive, and compact screening method that is convenient for use by paramedics in emergency medical services is needed.

**Figure 1:**
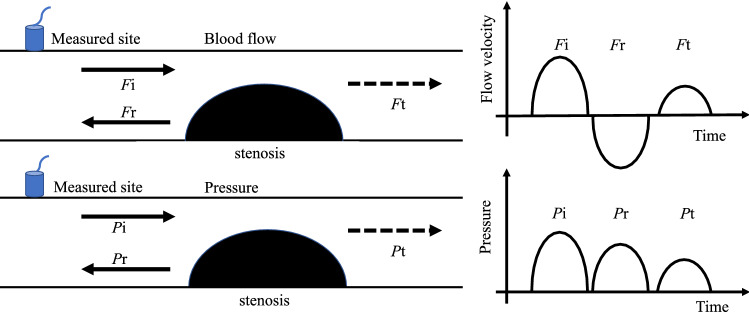
The concept of superposition of flow velocity and pressure waves near the stenosis. *F* and *P* imply the flow and pressure waves, respectively. The subscripts i, r, and t denote incident, reflected, and transmitted waves, respectively. A part of the transmitted wave reflects at the vascular bed in brain. If the actual pulse wavelength is very long, then, these waves generally superpose.

Considering the aforementioned aspects, we focus on simple pulse wave measurements to evaluate occlusion in the main artery of the anterior cerebral circulation in this study. The pulse wave is a temporal variation in the displacement of the skin surface caused by the pressure waves propagating in the artery. Thus, the pulse wave at the carotid artery is a superposition of incident and reflected waves^9^. The incident wave at the carotid artery is generated by a pressure wave and is referred to as the forward wave; it is caused by blood flow ejected from the heart. The reflected wave, referred to as the backward wave, is primarily generated by the reflection of the pressure wave from the vascular bed^10,11^. Acute ischemic stroke is generally caused by occlusion of a cerebral blood vessel by atherosclerosis or embolic thrombus. Such an occlusion also constitutes another reflector of the pulse waves, as shown in Fig. 1. These physical characteristics of pulse waves enable us to measure and analyze pulse waveforms and detect occlusion.

Measurement techniques that determine the pulse wave velocity (PWV) and cardio-ankle vascular index (CAVI) are widely used to evaluate arterial stiffness in the body. PWV is obtained from the time difference between two pulse waves measured at a known distance between two pressure sites in the arms and legs. As pulse waves propagate faster in stiff vessels, the measured value increases with the progression of atherosclerosis. However, PWV and CAVI devices measure pulse waves from pressure cuffs mounted on the human body and not those propagating to the brain. Another technique, the augmentation index (AI), is also used to evaluate arterial wall stiffness without occlusion. Similar to the method used to determine PWV, the AI is calculated using the observed pulse wave to extract factors from the peak values of the observed waves. However, pulse waveform analysis of AI does not incorporate various wave characteristics^12,13^.

In our previous study on the assessment of artery stiffness, we constructed a simple and inexpensive pulse-wave measurement device using a commercial piezoelectric sensor for ultrasonic ranging. This sensor could sense displacement at low frequencies, and in our previous study, we reported the in vivo pulse wave data measured in the carotid artery in healthy elderly individuals using this sensor^14^. We also compared the results obtained by pressure wave propagation in an artificial vascular model. The study indicated that pressure waves reflected at the vascular bed of the cerebral arteries could be perceived in the form of pulse waveforms at the carotid artery^15^. We envision that this device can be used to detect occlusions.

In this study, we focus on the reflection of the pulse wave at occlusion and propose a screening system to evaluate carotid artery occlusion using a simple pulse wave measurement technique. As occlusion rarely occurs simultaneously in bilateral cerebral arteries, we focus on the difference in pulse waveforms observed on both sides. To infer occlusion, we use a simple machine-learning technique with a few dynamic features extracted from pulse waveforms, which are only related to the reflection phenomenon at the occlusion. Here, other factors of the data are excluded to ensure the explainability and reliability of the outcome from a physical perspective. We then use a simple classifier for occlusion inference.

### Contribution of this work to pulse wave analysis

From the perspective of pulse wave analysis, readers of this paper may question the significance of this research. We would like to clarify the contribution of this work. Other studies have used pulse waves to assess cardiovascular diseases such as arterial stiffness^16–18^. To the best of our knowledge, these studies do not focus on inferring cerebral artery occlusion in emergencies. Our current study is the first attempt to address this issue using predictive machine learning.

Unlike conventional studies, we focus on the irregular, non-stationary change in the pulse wave caused by the superposition of waves reflected from an occlusion. Our idea is that the difference between left and right pulse waves contains crucial information about such changes and contributes to occlusion inference. The idea is simple yet novel, and it is embodied in the features we extract.

From a technical perspective, the classifier used in this study is the basic one, the logistic regression, not deep learning techniques. The reason for using such a classifier is to achieve both explainability and performance with limited data. The simplest classifier (in other words, a clear box) makes the effects of features explainable. It is often difficult to collect a large amount of data on occlusion patients, especially in emergencies. Furthermore, the position, size, and shape of occlusions vary greatly among patients, making repetitive measurements of similar symptoms challenging. Given these considerations, we selected a classifier that performs well even with limited data.

As a first attempt, this study makes a contribution to the inference of cerebral artery occlusion by demonstrating the fundamental effectiveness of the proposed method. Collecting more data and improving the classifier will be the focus of our next study.

### Ethical approval and availability of data and materials

All the experiments and data analyses in this study were performed in accordance with the Declaration of Helsinki. They were also approved by the medical ethics committees of Doshisha University (No. 18016, Mar. 5th, 2019) and Nara Medical University (No. 2111, Jan. 21st, 2019). Experiments and analyses were conducted properly following the guidelines set by the medical ethics committees, in addition to the guidelines set by our research group. The data generated/analyzed in this study are available from the corresponding author if the ethical committees of Doshisha university and Nara medical university allow the applicants to use the data. It is worth noting that none of the materials used in this study needed approval.

## Proposed method

**Figure 2:**
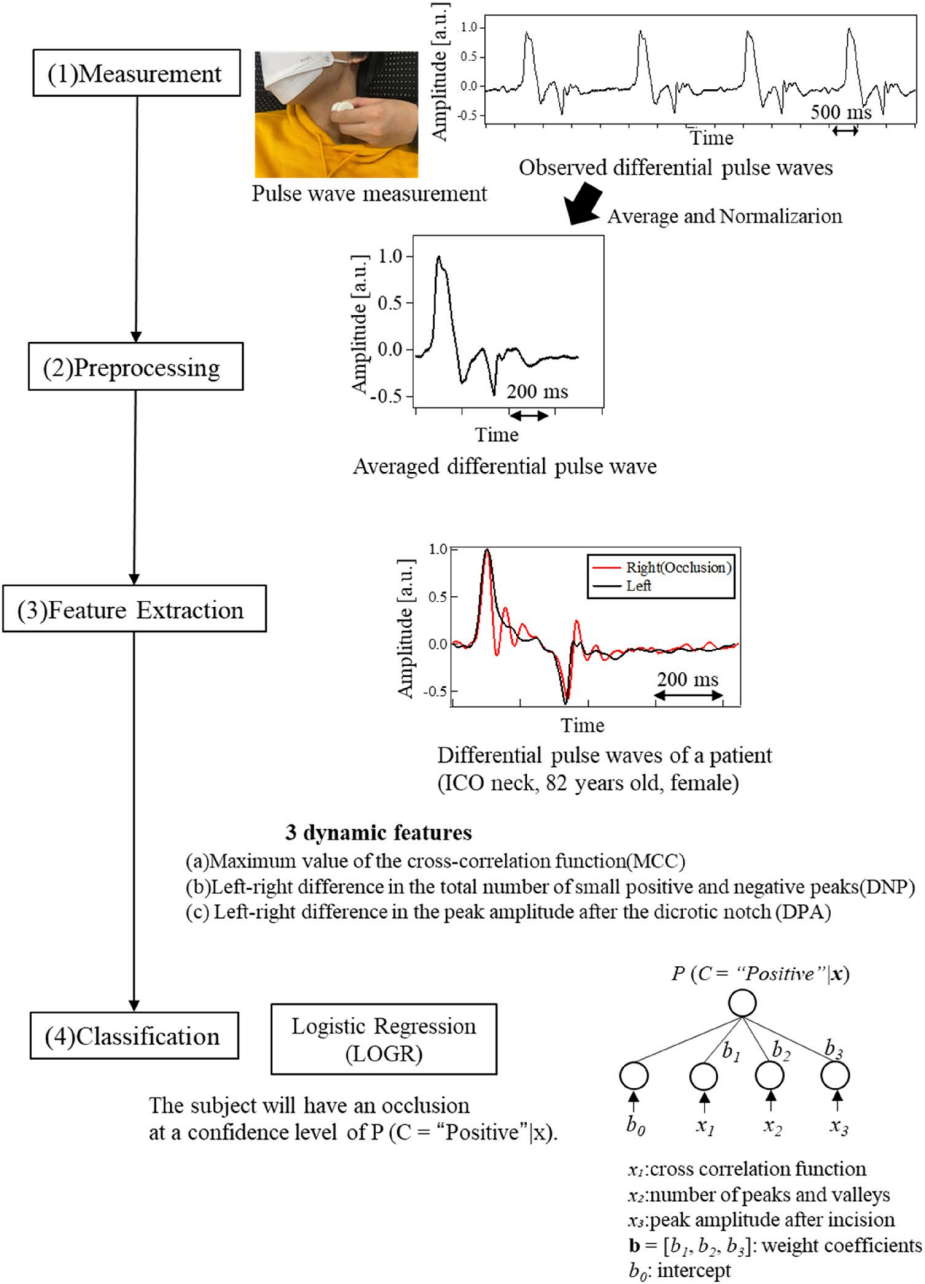
Procedures for the analysis of the measured pulse waves.

Typically, LVOS of anterior cerebral circulation is caused by unilateral artery occlusion. Therefore, for effective occlusion detection, we proposed pulse wave measurement in both the left and right carotid arteries of a person. The proposed method comprises three elemental ideas and techniques, namely, pulse wave measurement, feature extraction with preprocessing, and occlusion inference by classification. The procedures of the proposed method are illustrated in Fig. 2. The information on subjects selected for in vivo studies as well as the methodology that was followed is detailed in subsequent sections.

### Selection of subjects

In vivo studies were conducted on healthy subjects consisting of 15 men and 15 women in the age group of 20s to 90s. The subjects had no history of cardiovascular disease and were not consuming medication for hypertension. Among the patients, 16 men and 7 women with ages in the range of 51 to 96 exhibited an occlusion of the main cerebral artery in the anterior circulation. Table 1 lists ages and characteristics of the subjects. The stage of the disease was acute in 16 patients and chronic in 7 of them. Occlusion was diagnosed by digital subtraction angiography (DSA), computed tomography angiography (CTA), and magnetic resonance angiography (MRA). It was observed that 21 patients had internal carotid artery occlusion (ICO).

**Table 1:** Information of subjects.

Healthy (negative)		Healthy (negative)		Patients (positive)					
Age	Sex	Age	Sex	Age	Sex	Occlusion side	ICO (neck)	ICO (intra)	MCO
21	Male	54	Female	51	Male	R	1	–	–
21	Female	60	Male	58	Male	R	1	–	–
22	Male	61	Male	60	Male	L	1	–	–
22	Male	60	Male	63	Female	R	–	1	–
22	Female	62	Male	68	Male	L	1	–	–
22	Female	62	Female	70	Male	L	1	–	–
22	Female	63	Male	71	Male	L	1	–	–
23	Male	65	Female	72	Female	R	1	–	–
23	Female	67	Male	75	Female	L	1	–	–
23	Female	70	Female	78	Male	R	–	1	–
23	Female	70	Female	80	Male	R	1	–	–
41	Female	75	Male	82	Male	L	1	–	–
48	Female	82	Male	82	Female	R	1	–	–
50	Male	84	Male	82	Male	R	–	1	–
52	Male	90	Female	82	Female	R	1	–	–
–	–	–	–	83	Male	R	1	–	–
–	–	–	–	84	Male	L	1	–	–
–	–	–	–	84	Male	L	1	–	–
–	–	–	–	86	Female	L	–	–	1
–	–	–	–	87	Male	R	–	1	–
–	–	–	–	87	Male	L	–	–	1
–	–	–	–	89	Male	L	1	–	–
–	–	–	–	96	Female	R	–	1	–

Among them, 16 patients had extracranial ICO and 5 had intracranial occlusion, termed as ICO (neck) and ICO (intra) respectively. Furthermore, two patients had occlusion of the main trunk of the middle cerebral artery, termed as MCO. The point at which the common carotid artery branches into external and internal carotid arteries is termed as carotid bifurcation. An informed consent for the participation in the study was obtained from each participant.

### Pulse wave measurement

Occlusion has a significant effect on the characteristics of pulse waves at the carotid artery. However, these characteristics may also be influenced by several factors such as the condition of the patient. To understand the relationship between occlusion and the characteristics of pulse waves, other contributing factors were precluded from this study. For example, prior to commencing the measurements, all subjects avoided eating, exercising, and smoking for over 2 h. This was followed by resting in the supine position for 15 min in a quiet room at 25 °C. The cardiovascular function and vasomotor tone in the resting conditions were thus obtained^19,20^.

**Figure 3:**
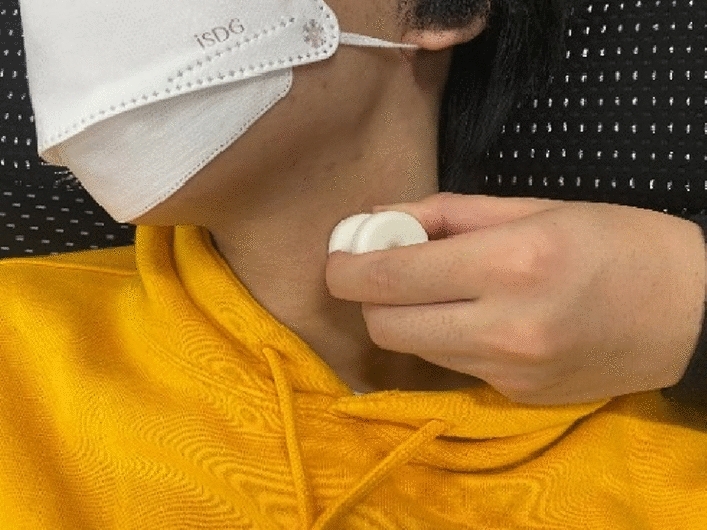
Pulse wave measurement.

The schematic of the measurement condition is illustrated in Fig. 3. The pulse wave was measured at the skin surface by placing a piezoelectric ceramic transducer (MA40E7R, Murata Corp.) at the upper edge of the thyroid cartilage, the position where the strongest pulse wave could be sensed by a finger. We measured pulse waves in both the right and left common carotid arteries. The observed signal was amplified by 40 dB using a preamplifier (NF 5307) and was subsequently digitized using a 14-bit analog-to-digital converter (Keyence NR-500, NR-HA08, or using our prototype measurement system manufactured in collaboration with Proassist. Ltd.) with a sampling frequency of 1.0 kHz^21^. In accordance with the characteristics of the sensor and the circuit system, the measured pulse wave corresponded to the differential (velocity) waveform in the low-frequency range. Thereafter, an average of the observed waves was obtained, and the DC component was eliminated to obtain the averaged differential pulse waveforms. Differential pulse waves (not integrated pulse waves) were consistently used in the measurement, feature extraction, and classification of the proposed method. Hereinafter, we use the word "pulse wave" to indicate "differential pulse wave" for simplicity.

### Feature extraction

The following text explains the preprocessing performed to acquire a single differential pulse wave using the raw time series data consisting of multiple waves. First, we selected 5–10 cycles from the stable portion of the differential pulse waves. In each cycle, a positive peak was followed by a negative peak. The heart-rate interval was calculated by measuring the time difference between the positive peaks. From the entire period, a section commencing 0.1 s prior to the positive peak was segmented from the raw time series and considered as a cycle. To reduce measurement noise, five such cycles were averaged and normalized, with the maximum amplitude of the positive peak set to one. This resulted in a single differential pulse wave for each subject. As mentioned earlier, occlusions in the main artery of the anterior circulation usually do not occur on both sides simultaneously. Therefore, we measured pulse waves in the left and right carotid arteries of patients with occlusion and healthy subjects. To infer occlusion, we extracted features that represent the difference between the left and right pulse waves. On the side with occlusion, blood flow is obstructed, causing the forward and reflected waves to overlap, resulting in the observed pulse wave. Based on this physical mechanism, we propose three dynamic features, MCC, DNP, and DPA. These features were extracted from differential pulse waves for better understanding of the dynamics.

#### Maximum value of the cross-correlation function (MCC)

The first of our proposed features *x*_1_ is the maximum value of the cross-correlation function. It is formulated as in Eq. (1). In this equation, *l*(*n*) and *r*(*n*) refer to the left and right differential pulse waves in which time *n* is discrete and ranges from 1 to N. *C*(*τ*) is the cross-correlation function parameterized by the shift time *τ*. The maximum of *C*(*τ*) is found through a search over the range *τ*. This is considered as the first feature, *x*_1_.

1 $$x_{1} = \mathop {\max }\limits_{\tau } C(\tau ) = \mathop {\max }\limits_{\tau } \frac{1}{N}\mathop \sum \limits_{n = 1}^{N} l(n)r(n + \tau )$$

#### Left–right difference in the total number of small positive and negative peaks (DNP)

The second proposed feature *x*_2_ is the absolute value of the difference in the number of small peaks between the left averaged differential pulse wave and the right one. This feature is formulated as in Eq. (2). The small peaks are ascertained between the first positive and last negative peaks. For instance, in the case of the left pulse wave, small negative and positive peaks are identified between the foremost positive and hindmost negative peaks. The condition for a peak is $\left(\frac{dl(t)}{dt}{}\right)_{t=n}=0$. The identified peaks are aggregated into the set *S*_*lp*_. for the left side. The same applies to *S*_*rp*_ for the right side. *#S* denotes the cardinality of set *S*, which is the number of set members. The absolute value of the subtraction of* #S*_*rp*_ from *#S*_*lp*_ yields the second feature, *x*_2_.

2 $$ \begin{aligned} x_{2} & = \left| {\# S_{lp} - \# S_{rp} } \right| \\ & = abs\left(\# \left\{ {l\left( n \right) | \left( {\frac{dl\left( t \right)}{{dt}}} \right)_{t = n} = 0} \right\} \right. \\ & \quad\left. - \# \left\{ {r\left( m \right) | \left( {\frac{dr\left( t \right)}{{dt}}} \right)_{t = m} = 0} \right\}\right) \\ \end{aligned} $$

#### Left–right difference in the peak amplitude after the dicrotic notch (DPA)

A dicrotic notch is the secondary upstroke caused by the closure of the aortic valve. It appears under the effect of the existence of occlusion. The strong negative peak in a differential pulse wave indicates the upcoming dicrotic notch. Hence, we focused on the positive peak adjacent to the last negative peak. The third proposed feature *x*_3_ is the absolute value of the difference between the amplitudes of this peak on the left and right sides. *x*_3_ is formulated in Eq. (3). *T*_np_ denotes the time of the last negative peak. The point *n* is identified based on the zero gradient $\left(\frac{dl\left( t \right)}{{dt}}\right)_{t = n} = 0$ and the maximum amplitude* l*(*n*) in the time segment ranged by *T*_np_. This point is regarded as the peak after the dicrotic notch in the left differential pulse wave. The points *m* and *r*(*m*) are determined similarly on the right side. The absolute value of the subtraction of* r*(*m*) from *l*(*n*) computes the third feature, *x*_3_.

3 $$ \begin{aligned} x_{3} & = \left| {l(n) - r(m)} \right| \\ & = abs \left(l(n) | \left( {\frac{dl(t)}{{dt}}} \right)_{t = n} = 0, \quad l(n) = \max l(i),\quad T_{np} \le i \right.\\ &\left. \quad - r(m) | \left( {\frac{dr(t)}{{dt}}} \right)_{t = m} = 0, \quad r(m) = \max r(j),\quad T_{np} \le j \right) \\ \end{aligned} $$

**Figure 4:**
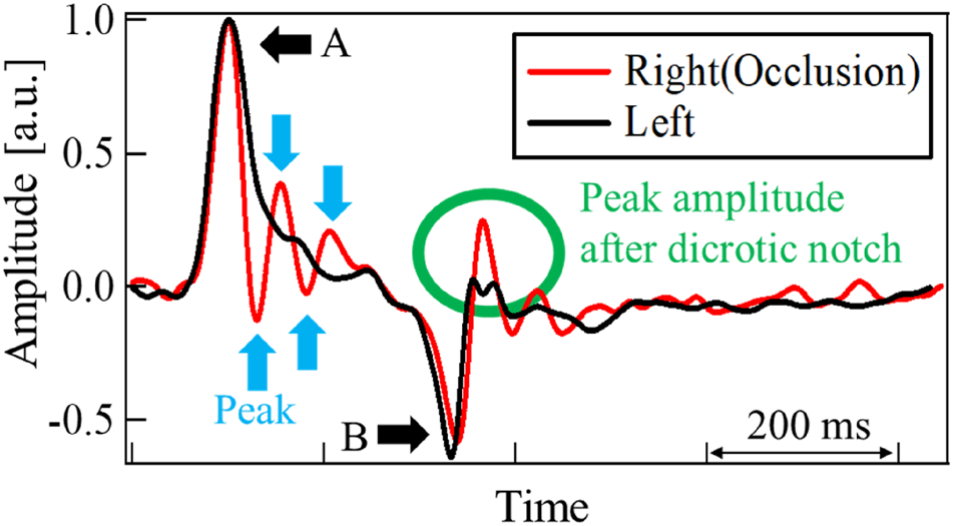
Differential pulse waves of patient (ICO neck, 82 years old, female). A: positive peak, B: negative peak. Between A and B, small peaks were observed.

Figure 4 depicts the differential pulse waveform of a patient with occlusion. Here, the points A and B in the waveform illustrate the first positive and last negative peaks, respectively. The small positive and negative peaks between them are also indicated using arrows. The three dynamic features represent the different aspects of a pulse waveform. MCC focuses on the similarity of the entire waveform whereas DNP computes the internal reflection of pressure waves that occur when the heart ejects blood. DPA measures the blood flow velocity at the end of the diastole.

### Occlusion inference

As an initial attempt to infer occlusion, we applied the logistic regression (LOGR) to our three dynamic features^22^. LOGR is a fundamental machine learning (ML) technique that is widely used for classification. Compared to advanced ML techniques, the inference result of LOGR is easy to understand as the original features are not transformed in a complex way. Due to its simplicity, LOGR does not require large datasets or complex model selection using a validation set.

In general, LOGR assumes a linear regression function for each class *C*_*k*_, where *k* = 1, 2, …, K. By constraining the function values to a range between 0 to 1, LOGR estimates the conditional probability *P*(*C*_*k*_|**x**) that an instance **x** belongs to class *C*_*k*_. Then it classifies **x** to *C*_*k*_, which has the highest probability among all the classes. The model is trained using a training set to maximize its objective function, which is the log-likelihood. Finally, the performance of the LOGR model is evaluated using a test set to ensure generalizability.

While the above is a multiclass classification task, the present study aims to infer the existence of occlusion, which is a binary class classification task. Therefore, we formulated LOGR with one class for the latter task. The input is a feature vector consisting of the three dynamic features for a subject, i.e., **x** = [*x*_*1*_, *x*_*2*_, *x*_*3*_]. A linear regression function for class *C* has the following two parameters to be optimized: the intercept *b*_*0*_ and weight vector consisting of the weight coefficients **b** = [*b*_*1*_, *b*_*2*_, *b*_*3*_] corresponding to *x*_*1*_, *x*_*2*_, and *x*_*3*_, respectively. By substituting this function into a sigmoid function, the conditional probability *P* (*C* = “*Positive*”*|****x***) is estimated using Eq. (4). This signifies that the subject will have an occlusion at a confidence level of *P* (*C* = “*Positive*”*|****x***). Consequently, 1 − *P* (*C* = “*Positive*”*|****x***) is the probability of “Negative, ” meaning that no occlusion exists at this level.

4 $$P(C = {\text{"}}Positive{\text{"}}|{\varvec{x}}) = \frac{1}{{1 + \exp \left(-( {b_{0} + {\varvec{b}} \cdot {\varvec{x}}} \right))}}.$$

After training LOGR to optimize *b*_0_ and ***b***, it can accurately estimate *P*(*C* = “*Positive*”*|****x***). LOGR classifies the subject as “Positive” if *P*(*C* = “*Positive*”|***x***) is greater than a preset threshold value. In our experiments, the ratio of positive subjects was 23/(23 + 30) = 0.43 (refer to Table 1). This value of 0.43 is the chance level, achieved by assuming that all the subjects are positive, and serves as the baseline performance. To avoid excessive false positives, the threshold of *P*(*C* = “*Positive*”|***x***) is set to 0.50, which is higher than the baseline value and is appropriate for a binary classification task.

To ensure an unbiased performance estimation, we designed the estimation process as follows: the data points in the original dataset were randomly shuffled and then split into a training set and a test set. In the experiments discussed later, the sizes of the training and test sets were 40 and 13, respectively. A LOGR model was created for the training dataset, and its performance is estimated using the test dataset. This set of procedures was repeated under different data randomizations 20 times. The performance of occlusion inference and the weights of the features were estimated each time and the average performance was also calculated.

## Result and discussion

### Measured pulse waves

**Figure 5:**
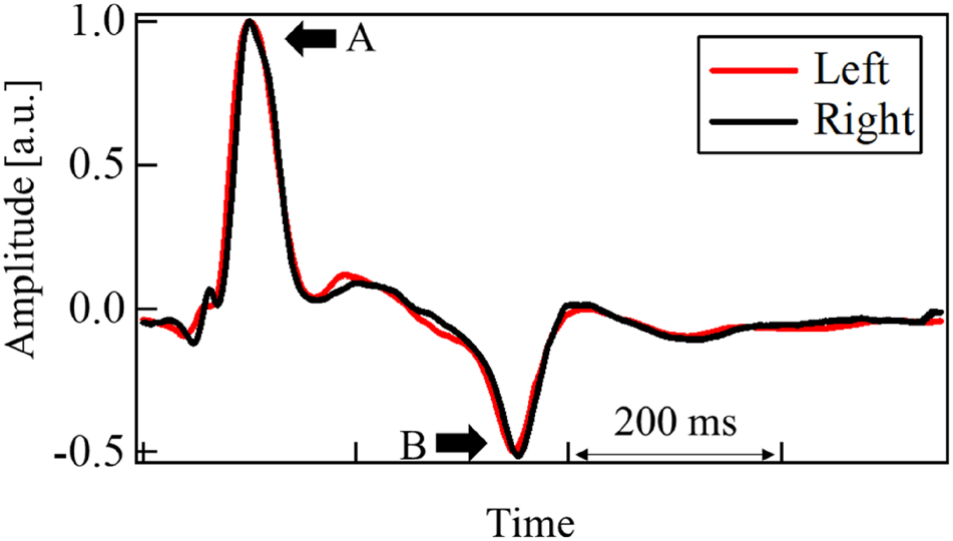
Differential pulse waves of healthy subject (54 years old, female). A: positive peak, and B: negative peak.

Figures 4 and 5 illustrate typical examples of differential pulse waveforms at the common carotid arteries of patients with occlusion and healthy subjects. Waveforms were normalized using their positive, and maximum peak values. For healthy subjects, the differential pulse waveforms on the left and right sides were similar. As mentioned above, pulse waveforms are affected by various factors such as standing/sitting position, blood pressure, and artery stiffness. These factors affect both left and right pulse waveforms but can be disregarded when comparing characteristic features of left and right waveforms. In contrast, in patients with occlusion, the left and right waveforms are often dissimilar because of occlusion. That brings the advantage of our proposal to utilize the left and right difference. Pulse waves measured at the carotid artery include reflected waves from the end of the cerebral arteries and vascular bed^7–9^. In the case of an occlusion in the cerebral artery, the wave reflected from the occlusion will be superposed at the carotid artery^23^. Shimada et al. reported that reflected waves from occlusion in smaller arteries in the brain may be observed at the carotid artery using an artificial cerebral model^24^. In the event of an occlusion close to the measurement site of the carotid artery, the reflected pressure wave may be observed clearly. This reflection is pronounced and appears earlier than that from the vascular bed. Taking the above knowledge and findings into account, the results for each feature are discussed in the following sections.

### MCC

**Figure 6:**
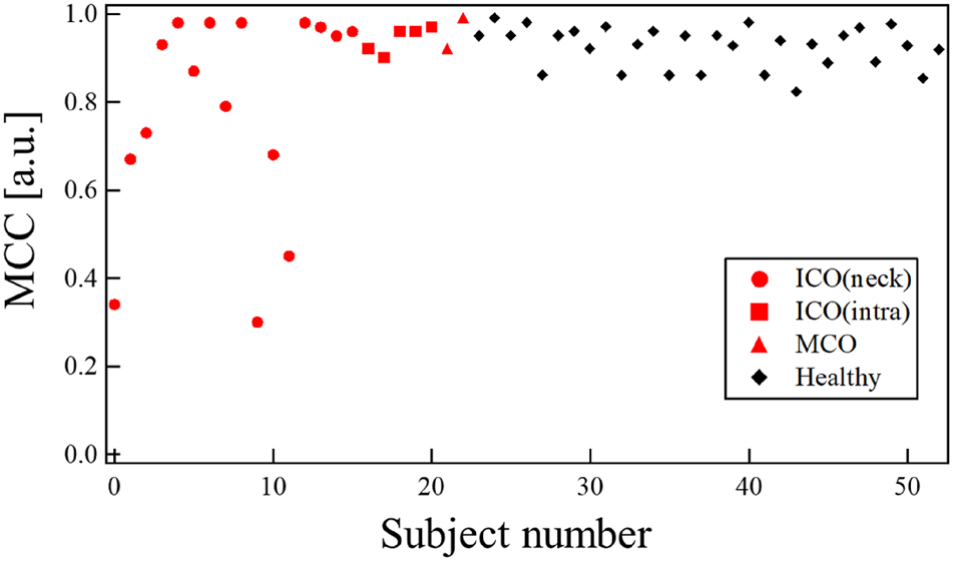
MCC for each subject.

In some cases, differences in the left- and right-side waveforms were clearly observed in patients with occlusions. Being the first proposed feature that represents the left and right differences, the MCC of each subject was estimated. The results are depicted in Fig. 6. The mean of MCC and their standard deviation (SD) were 0.83 and 0.21, respectively for the patient group with occlusion. Those were 0.92 and 0.05, respectively for the healthy group. Some patients with occlusion had values that were much smaller than the mean value. They were found to be afflicted with ICO (neck). The mean and SD for ICO (neck) were 0.78 and 0.22. The corresponding values for ICO (intra) and MCO patients were 0.94, 0.03, 0.96 and 0.04, respectively. Considering the average values, the cross-correlation was deemed as a good feature to select an ICO occlusion. As the ICO (neck) was closer to the measurement point, the reflection from the occlusion returned clearly. Additionally, owing to the MCC traversing at the entire pulse waveform, the shape of the positive and negative peaks was found to be an influencing factor. These peaks are represented as A and B in the waveforms shown in Fig. 4. It was perceived that this aspect could potentially lead to missing the effects of small variations in between these peaks.

### DNP

**Figure 7:**
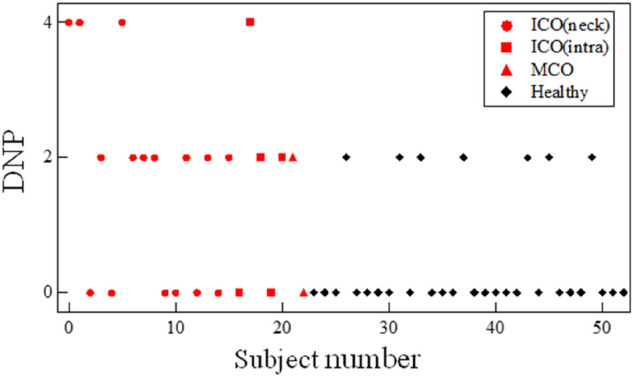
DNP for each subject.

To focus on the small change due to reflection, we calculated the difference in the number of small positive and negative peaks as the second proposed feature. As indicated by the arrows in Fig. 4, small peaks were observed only on the occlusion side. These resulted from repetitive reflections occurring between the occlusion site and the heart. The results for all the subjects are shown in Fig. 7. The DNP appeared larger in patients with occlusion than in all healthy subjects.

### DPA

**Figure 8:**
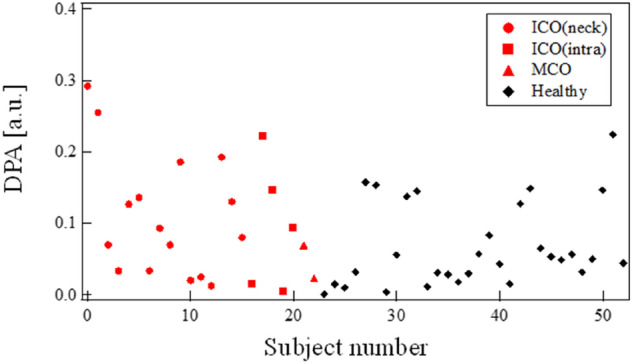
DPA for each subject.

The third feature of our proposed method is the DPA. As expected, this difference was observed in the differential pulse waveforms of the majority of the patients. The peak amplitudes after the dicrotic notch of the averaged differential pulse waves were often larger on the occlusion side than on the unaffected side. The results for all subjects are shown in Fig. 8. Yasaka et al. reported that the end-diastolic velocity of the unaffected side was faster than that of the affected side in the ICO and MCO groups^25,26^. In the occlusion group, the average and SD of DPA were 0.10 and 0.08, respectively. In healthy subjects, the mean and SD were 0.07 and 0.06, respectively. The DPA of the patients with occlusion was slightly larger than that of the healthy subjects. We consider that the end-diastolic blood flow velocity influences the DPA. The peak amplitude after the dicrotic notch of the affected side was larger than that of the unaffected side. Yasaka reported that the blood flow velocity around the occlusion is small on the affected side^25^. The low velocity indicates reflection of pressure at the occlusion, which is consistent with our DPA data.

### Accuracy of inference by logistic regression

We examined the independence of the three features and the results are presented in Table 2. To determine the independence, we calculated the correlation between each feature. As we focused on different parts of the differential pulse waveform, a weak correlation was observed between the features. The experimental results suggest that the left–right difference is caused by the influence of the reflection from the occlusion site. However, the pulse waves in both sides also include reflections from the vascular bed. These reflections may result in an unexpectedly weaker correlation between each feature and the existence of occlusion. Although each feature does not have a high correlation with the existence of occlusion, it is expected that the three features complement each other and contribute to occlusion inference as a whole. This is confirmed in the next paragraph.

**Table 2:** Correlations for each combination of the three dynamic features and the existence of occlusion for magnetic properties.

	MCC	DNP	DPA	*C*
MCC	–	− 0.25	− 0.43	− 0.30
DNP	–	–	0.54	0.44
DPA	–	–	–	0.33

**Figure 9:**
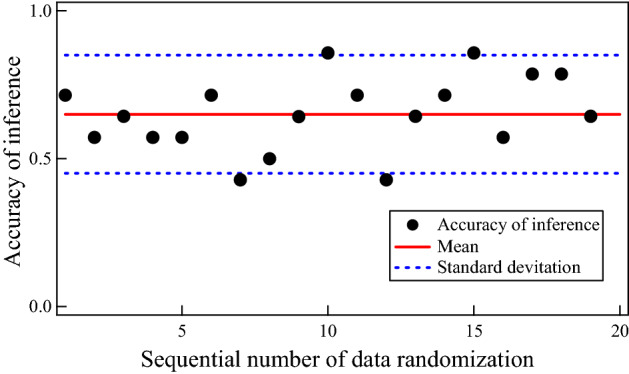
Accuracies of inference of a logistic regression model for a test dataset under different data randomizations.

By applying LOGR, we obtained the accuracies of the proposed method for a test dataset in trials with randomized data orders. Note that accuracy is the ratio of correct inferences for both positive and negative subjects to all subjects. The definition of accuracy is given in Eq. (5), where TP, FP, TN, and FN are the numbers of true positives, false positives, true negatives, and false negatives, respectively^27^. As shown in Fig. 9, the accuracy fluctuated depending on the randomization, so we calculated its mean and SD. We also obtained the inference performances for positive subjects, precision and recall, as it is important to correctly and completely detect existing occlusions. Precision is the ratio of correct inferences for positive subjects to subjects inferred to be positive, and is defined in Eq. (6). Recall, defined in Eq. (7), is the ratio of those to subjects that actually have an occlusion. Table 3 summarizes the means and SDs of accuracy, precision, and recall.

5 $$Accuracy = \frac{TP + TN}{{TP + FP + TN + FN}}.$$

6 $$Precision = \frac{TP}{{TP + FP}}.$$

7 $$Recall = \frac{TP}{{TP + FN}}.$$

**Table 3:** Performances of occlusion inference. The mean and standard deviation are provided for each of accuracy, precision, and recall. **Indicates that the accuracy is statistically higher than the chance level at the significant level of 0.01.

	Accuracy	Precision	Recall
Mean	0.65**	0.65	0.53
Standard deviation	0.20	0.20	0.20

The mean of accuracy was 0.65, which was statistically higher than the chance level of 0.43 with p < 0.01, as determined by the *t* test. The mean precision was 0.65, and the mean recall was 0.53 as shown in Table 3. While the inference performance for negative subjects is not the primary focus, it must be considered in terms of clinical practicality. Therefore, we also calculated the mean and SD of the negative predictive value (NPV), which were 0.69 and 0.18 respectively. In general, there is a trade-off among precision, recall, and NPV. If one of them is extremely low, the others become extremely high, leading to a false high accuracy. In contrast, the proposed method achieved a balance of precision, recall, NPV, and accuracy. Overall, it was confirmed that the proposed method, including the three dynamic features and the classifier LOGR, worked well to some extent in inferring the existence of occlusion.

**Figure 10:**
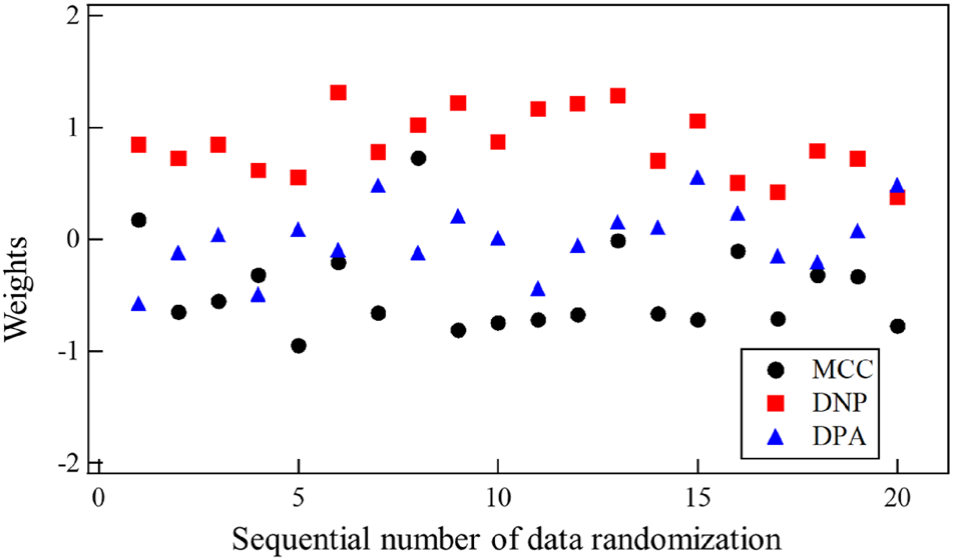
Weights on the three dynamic features, which were obtained via training a logistic regression model under different data randomizations.

Let us focus on the importance of the three dynamic features MCC, DNP, and DPA. Individually, they did not have a strong correlation with the positive class C (see Table 2), but their combination was effective in occlusion inference. To identify the importance of each feature (in other words, how much it contributed to occlusion inference), we investigated the weights given by LOGR. Figure 10 plots the weights in the trials with different random data orders and Table 4 provides the mean and SD of weights for each of MCC, DNP, and DPA.

**Table 4:** Final weights on the three dynamic features, which were obtained via training a logistic regression model under different data randomizations.

	MCC	DNP	DPA
Mean	0.45	0.85	0.21
Standard deviation	0.40	0.28	0.19

The mean weight of DNP (0.85) was the largest and significantly larger than 0.00. It is worth noting that DNP is the left–right difference in the number of small peaks, counted in the area between the first positive and last negative peaks of the differential pulse waveform. The large weight of DNP suggests that small fluctuations in the waveforms due to reflections are sufficiently represented by DNP and are important for occlusion inference. The mean weight of MCC 0.45 was the second largest. MCC is the cross-correlation representing the left–right difference in the entire waveforms, suggesting that such a global feature is also informative about occlusion. The mean weight of DPA 0.21 was not so large, but still had some contribution to occlusion inference. DPA is the left–right difference in the peak amplitude after the dicrotic notch. In the present experiment, the high importance of DPA was not suggested.

Here is the summary of results and discussion. It was experimentally confirmed that the proposed method, consisting of pulse wave measurement, feature extraction, and classification, can infer the existence of cerebral artery occlusion. The proposed method provided an explainable way of understanding how certain features contribute to the inference. Although its inference performance was not perfect, it could achieve results even with a small amount of data.

In this study, we focused on the effects of the reflected wave at the occlusion on the pulse wave observed at the carotid artery, resulting in differences between the left and right pulse waveforms. In the future, it will be important to consider the effects of pulse waveform changes due to age; adding data of both healthy and patient individuals aged 40–90 years will improve the study.

Additionally, owing to the electrical nature of the measurement system, the current system measures the differential waves of the pulse wave. With more careful integration of observed data, future studies of the pulse wave may provide additional information such as wave amplitude, which depends on the reflection condition at the occlusion.

## Conclusion

Toward the ultimate goal of establishing an occlusion diagnosis support system in emergency medical services, we proposed a method that included a noninvasive measurement of both left and right pulse waves at the carotid artery using a piezoelectric sensor system, the three dynamic features extracted from these pulse waves, and the occlusion inference using these features. In the experiments, we measured both left and right carotid artery pulse waveforms of patients with occlusion as well as healthy subjects. We then extracted the cross-correlation between the left and right waveforms, the number of small positive and negative peaks, and the left and right differences in peak amplitude after dicrotic notch. By applying the logistic regression to these features, we inferred the existence of occlusion. Finally, the accuracy of occlusion inference was estimated as 0.65, which was higher than the chance level of 0.43. This study utilized the logistic regression, which is one of the basic machine learning methods, to comprehend the effect of each feature. As mentioned above, this study focused on three simple features which only resulted from the reflection phenomenon at the occlusion. In the forthcoming research, the accuracy of the occlusion inference will be improved by employing high performance machine learning methods, using other features of waveforms, and information of each data such as age, sex etc. To further enhance the accuracy, we plan to increase the number of subjects and identify the most suitable dynamic features for detecting occlusion. It is expected that our present system will work well not only for occlusion inference but also for screening other diseases with vascular deformation, such as aneurysms.
